# Whole Genome Sequence of *Lactiplantibacillus plantarum* HOM3204 and Its Antioxidant Effect on D-Galactose-Induced Aging in Mice

**DOI:** 10.4014/jmb.2209.09021

**Published:** 2023-06-02

**Authors:** Di Zhang, Heesung Shin, Tingting Wang, Yaxin Zhao, Suwon Lee, Chongyoon Lim, Shiqi Zhang

**Affiliations:** 1 Coree Beijing Co., Ltd., No. A-7 Tianzhu West Rd., Tianzhu Airport Industrial Zone A, Shunyi District, Beijing 101312, P.R. China; 2 Dx&Vx Co., Ltd., Seoul 13201, Republic of Korea; 3 Health Food Function Testing Center, College of Applied Arts and Science, Beijing Union University, Beijing 100101, P.R. China

**Keywords:** *Lactiplantibacillus plantarum*, whole genome sequence, antioxidant activity, D-galactose-induced aging, oxidative stress

## Abstract

*Lactiplantibacillus plantarum*, previously named *Lactobacillus plantarum*, is a facultative, homofermentative lactic acid bacterium widely distributed in nature. Several *Lpb. plantarum* strains have been demonstrated to possess good probiotic properties, and *Lpb. plantarum* HOM3204 is a potential probiotic strain isolated from homemade pickled cabbage plants. In this study, whole-genome sequencing was performed to acquire genetic information and predict the function of HOM3204, which has a circular chromosome of 3,232,697 bp and two plasmids of 48,573 and 17,060 bp, respectively. Moreover, various oxidative stress-related genes were identified in the strain, and its antioxidant activity was evaluated in vitro and in vivo. Compared to reference strains, the intracellular cell-free extracts of *Lpb. plantarum* HOM3204 at a dose of 10^10^ colony-forming units (CFU)/ml in vitro exhibited stronger antioxidant properties, such as total antioxidant activity, 2,2-diphenyl-1-picrylhydrazyl radical scavenging rate, superoxide dismutase activity, and glutathione (GSH) content. Daily administration of 10^9^ CFU *Lpb. plantarum* HOM3204 for 45 days significantly improved the antioxidant function by increasing the glutathione peroxidase activity in the whole blood and GSH concentration in the livers of D-galactose-induced aging mice. These results suggest that *Lpb. plantarum* HOM3204 can potentially be used as a food ingredient with good antioxidant properties.

## Introduction

Probiotics are live microorganisms that confer health benefits to the host when administered in adequate amounts [1]. *Lactiplantibacillus plantarum* is one of the main research topics in the field of probiotics. It is a facultative, homofermentative lactic acid bacterium that is widely distributed in fermented foods and various ecological niches, including plants, animals, and the human gut [2, 3]. According to the Food and Agriculture Organization of the United Nations/World Health Organization, probiotics should have outstanding gastrointestinal tolerance, intestinal epithelial cell adhesion ability, and safety [4]. *Lpb. plantarum* strains exhibit good acid and bile salt tolerance and have various beneficial effects on the host, such as the regulation of intestinal flora [5] and immune response [6], increase in antioxidant activity [7], and reduction of cholesterol and glucose levels [8]. In a previous study, we isolated a *Lpb. plantarum* strain, HOM3204, from homemade pickled cabbage [9]. In vitro, *Lpb. plantarum* HOM3204 showed strong tolerance to simulated gastric and intestinal juice, high adhesion to Caco-2 cells, and good antimicrobial activity [9]. It significantly recovered the intestinal flora in ampicillin-induced dysbiotic mice by decreasing the abundance of *Enterococci*, while increasing the abundance of *Lactobacilli* and *Bifidobacterium*. The strain also enhanced the antioxidant capacity by increasing the levels of glutathione peroxidase (GSH-Px) and superoxide dismutase (SOD) in the serum [9].

The requirement of whole-genome sequencing (WGS) analysis of probiotic candidates to assess their food safety was proposed by the European Food Safety Authority in 2019 [10]. Accordingly, genes encoding antimicrobial resistance, virulence, and toxigenicity were subjected to extensive assessments [10]. Whole genome sequences of many *Lpb. plantarum* strains were sequenced and deposited in the GenBank sequence database to better understand and explore their probiotic functions (https://www.ncbi.nlm.nih.gov/genome). *Lpb. plantarum* WCFS1 is the first *Lpb. plantarum* strain that was completely genome sequenced [11]. Jia *et al*. demonstrated the whole genome sequence of *Lpb. plantarum* KLDS1.0391 and its good adhesion performance in their study [12]. Kwak *et al*. reported the whole genome sequence of *Lpb. plantarum* GB-LP2 and its enhanced immune properties [13].

Reactive oxygen species (ROS), including hydroxyl radicals, superoxide anions, and hydrogen peroxide, are produced via oxygen metabolism and balanced by the rate of oxidant formation and elimination [14, 15]. Oxidative stress, caused by an imbalance between the generation of ROS and antioxidant defense systems, is associated with the natural aging process and pathogenesis of many diseases [16]. Accumulating evidence demonstrates that probiotics are effective against oxidative stress via enzymatic antioxidant defenses, including SOD, GSH-Px, and glutathione reductase (GR), and antioxidant metabolites, such as GSH, butyrate, and folate [17, 18]. Several *Lpb. plantarum* strains have been proven to possess good antioxidant properties [19, 20].

The D-galactose-induced aging mouse model, which mimics natural aging, is one of the most commonly used models for oxidative stress studies [21]. Researchers often employ this model to determine the anti-aging activities and antioxidant effects of probiotics [21-23]. D-Galactose injection increases oxidative stress by increasing the malonaldehyde (MDA) levels and decreasing the activity of antioxidant enzymes in mice [24, 25]. MDA is the principal and most studied product of polyunsaturated fatty acid peroxidation [26]. Some studies have assessed MDA to quantify the level of oxidative stress in vitro and in vivo [26].

In the present study, we conducted WGS analysis of *Lpb. plantarum* HOM3204 and determined its antioxidant activity in vitro. The D-galactose-induced aging mouse model was selected to preliminarily evaluate the ability of *Lpb. plantarum* HOM3204 to cope with oxidative stress via enzymatic and non-enzymatic defenses in mice.

## Materials and Methods

### Genomic DNA Extraction, Genome Sequencing, Assembly, and Annotation

The whole genome of *Lpb. plantarum* HOM3204 was sequenced by OE Biotech (China) using the shotgun strategy. Genomic DNA was extracted using a Bacterial DNA Kit D3350 (Omega, USA). DNA was quantified using a NanoDrop spectrophotometer (Thermo Fisher Scientific, USA) and Qubit (Thermo Fisher Scientific) and subjected to agarose gel electrophoresis.

The genome was sequenced using the PacBio Sequel (Pacific Biosciences, USA)  and Illumina HiSeq platforms (Illumina Inc., USA) [27]. Low-quality reads were filtered out using the single-molecule, real-time sequencing technology (SMRT, v2.3.0) and the high-quality filtered reads were assembled to generate one contig without any gaps [28]. The paired-end strategy was used in the Illumina sequencing platform. Falcon (v0.3.0) was used for sub-read self-correction and three-generation sequence assembly [29]. Sub-reads were then processed to generate consensus sequences using Quiver (v2.2.2) [28]. A single-pass read accuracy improver (Sprai, v0.9.9.23) was used to correct the sequencing errors in single-pass reads [30]. Contigs were circularized using Circlator [31]. The assembled genome was annotated to identify the protein-coding and RNA genes using the National Center for Biotechnology Information (NCBI) Prokaryotic Genome Annotation Pipeline [32].

Gene prediction of the assembled genome was conducted using Prodigal (v2.6.3) [33]. Functions of the predicted protein-coding genes were annotated using the Clusters of Orthologous Groups (COG) database annotations based on protein alignment using the Diamond software (e-value < 1e^-5^) [34]. Prophages were predicted using PhiSpy (v2.3) [35]. Pathogen–host interactions and the Comprehensive Antibiotic Resistance Database (CARD) were used for pathogenicity and drug resistance analyses, respectively [36, 37]. Carbohydrate-Active Enzymes (CAZy) analysis was performed using the CAZy database [38]. tRNA and rRNA genes were predicted using tRNAscan-SE (v1.3.1) [39] and rRNAmmer (v1.2) [40], respectively. Finally, sRNAs were predicted using BLAST against the Rfam database [41], and the circular genome graph was created using Circos (v0.69) [42].

### Comparative Analysis

Ten reference *Lpb. plantarum* complete genomes were downloaded from the NCBI and European Nucleotide Archive databases. The accession numbers were CP021997.1 (LPL-1), CP004082.1 (ZJ316), CP005942.2 (P-8), CP006033.1 (16), CP019348.1 (KLDS1.0391), GCA_001888735 (299v), CP002222.1 (ST-III), CP033616.1 (J26), CP017066.1 (LP3), and AL935263.2 (WCFS1). The average nucleotide identity (ANI) tree of 10 *Lpb. plantarum* strains and the *Lpb. plantarum* HOM3204 strain was constructed using Pyani software [43]. A phylogenetic tree was used to describe the evolutionary relationships between the strains based on WGS data. ParaAT (v2.0) was used as a parallel tool for constructing multiple protein-coding DNA alignments [44], and a maximum likelihood (ML) phylogenetic tree was constructed using RAxML [45].

### Evaluation of Antioxidant Activity of *Lpb. plantarum* Strains In Vitro

*Lpb. plantarum* Lp-115 and *Lpb. plantarum* ST-III were isolated from a solid beverage (Dupont, USA) and a fermented milk drink (Bright Dairy, China), respectively, and are reference strains which are popular on the market. *Lpb. plantarum* strains (HOM3204, Lp-115, and ST-III) were aerobically cultivated thrice in the de Man, Rogosa and Sharp broth at 37°C for 24 h. Bacterial cells were harvested via centrifugation (11,000 ×*g*, 10 min), washed thrice with phosphate-buffered saline (PBS), and resuspended in PBS with a viable cell density of 4 × 10^10^ colony-forming units (CFU)/ml. To obtain intracellular cell-free extracts, the suspension of intact cells was disrupted using a homogenizer (APV1000; SPX, Germany) at 850 bar for 10 min. Debris was removed via centrifugation (11,000 ×*g*, 10 min).

T-AOC and hydroxyl radical scavenging, SOD, GSH-Px, and GSH activities were determined using A015, A018-1-1, A001-2, A005, and A006-1-1 assay kits, respectively (China). Following this, 1,1-diphenyl-2-picrylhydrazyl (DPPH) radical scavenging activity was determined, according to a modified method of Lin and Chang [46]. Briefly, 2 ml of intracellular cell-free extract was mixed with 2 ml of DPPH ethanol solution (0.2 mmol/l). The mixed solution was placed in the dark for 30 min at 25°C and centrifuged at 11,000 ×*g* for 10 min to obtain the supernatant. The absorbance of the supernatant was measured at 517 nm using a spectrophotometer (UV-1800; Shimadzu, Japan) and marked as Ai. For the blank control, the DPPH ethanol solution was replaced with an equal volume of ethanol and the absorbance was marked as Aj; the sample solution was replaced with an equal volume of distilled water and the absorbance was marked as A0; and a mixture of distilled water and ethanol solution was used to adjust the absorbance to zero. Ascorbic acid (0.5 mg/ml; Sigma-Aldrich, USA) was used as the positive control. DPPH scavenging activity was calculated using the following equation:

DPPH scavenging activity (%) = [1 – (Ai – Aj)/A0] ×100%.

All in vitro assays were performed in triplicates.

### Antioxidant Effects of *Lpb. plantarum* HOM3204 on D-Galactose-Induced Aging in Mice

Freeze-dried *Lpb. plantarum* HOM3204 bacterial powder was produced according to a previously described method [9]. Thirty specific-pathogen-free (SPF), male KM mice (22–26 g, 8-week-old) were purchased from Beijing HFK Bioscience Co., Ltd. (China). The animal experimental protocol was approved by the Ethics Committee of the Health Food Function Testing Center, College of Applied Arts and Science, Beijing Union University (No. 2020-02). The feeding environment of mice was maintained at 22 ± 2°C and 55 ± 5% humidity. Five mice were raised in a cage and fed a pathogen-free diet and water under a 12/12 h light/dark cycle. All materials were autoclaved before use. After one-week of adaptation, 20 mice were subcutaneously injected with 300 mg of D-galactose/kg of body weight for six weeks to establish the D-galactose oxidative damage model. The residual 10 mice were assigned to the control group and subcutaneously injected with an equal volume of sterile deionized water. Each group had 10 mice. The D-galactose oxidative damage model was successfully established, and the MDA level in this model was significantly increased compared to that in the control group (*p* < 0.01). Twenty mice belonging to the D-galactose oxidative damage model were randomly divided into the model and *Lpb. plantarum* HOM3204 groups. The *Lpb. plantarum* HOM3204 group was orally administered with the *Lpb. plantarum* HOM3204 powder (1 × 10^9^ CFU, once daily) for 45 days, and the model and control groups were orally administered with sterile deionized water. Meanwhile, the model and *Lpb. plantarum* HOM3204 groups were injected with the same dose of D-galactose over a 45-day period. The control group was injected with an equal volume of sterile deionized water. Body weights were measured on days 0 and 45. Twenty-four hours after the final gavage, the eyeball blood was collected to determine the GSH-Px levels. The serum of the eyeball was used to measure the MDA and SOD levels. GSH and protein carbonyl levels in the liver were also measured. All indices were determined according to the instructions of the assay kits (China).

### Statistical Analysis

Data are presented as the mean ± standard error of the mean. Data analysis was conducted using one-way analysis of variance, followed by Tukey’s multiple comparisons test with the SPSS software (version 25, IBM, Corp., USA). Values were considered statistically significant at *p* < 0.05.

## Results

### Genome Features

**Fig. 1:**
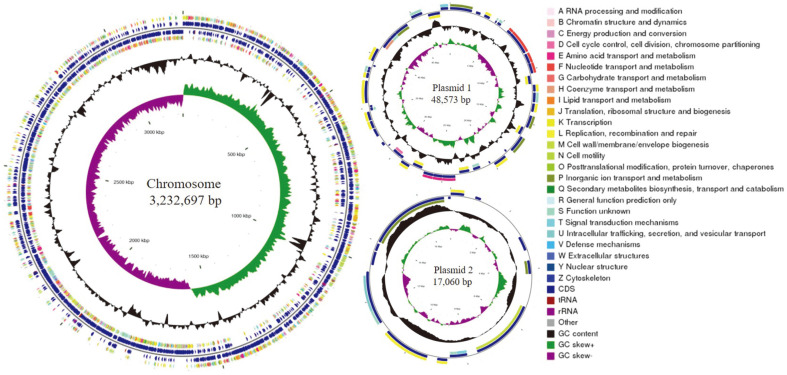
Circular genome graph of *Lactiplantibacillus plantarum* HOM3204. Circles, from inside to outside, represent the genome size, GC skew, GC contents, coding sequence (CDS) in the reverse strand, tRNA and rRNA genes in reverse strand, tRNA and rRNA genes in forward strand, and CDS in forward strand. A–Z, respectively, indicate the functional classification of CDS genes on the chromosome and plasmids using the Clusters of Orthologous Groups (COG) database. Circos (v0.69) software was used to create a genomic map with the given information.

As shown in Fig. 1 and Table 1, the complete genome of *Lpb. plantarum* HOM3204 was composed of one circular chromosome (3.23 Mbp) with a GC content of 44.61% and two circular plasmids (plasmid 1 [48,573 bp] with 39.04% GC content and plasmid 2 [17,060 bp] with 40.57% GC content). There were 3,027 genes, 122 RNA genes (16 rRNA, 68 tRNA, and 38 sRNA genes) in the circular chromosome, three RNA genes (0 rRNA, 0 tRNA, and 3 sRNA genes) and one RNA gene (0 rRNA, 0 tRNA, and 1 sRNA gene) in plasmid 1 and plasmid 2, respectively.

One prophage in plasmid 1 was identified using PhiSpy. No drug resistance and virulence genes were found according to the minimum cutoff of 90% nucleotide identity over a minimum coverage length of 60% [47, 48] using CARD and VFDB, respectively.

**Fig. 2:**
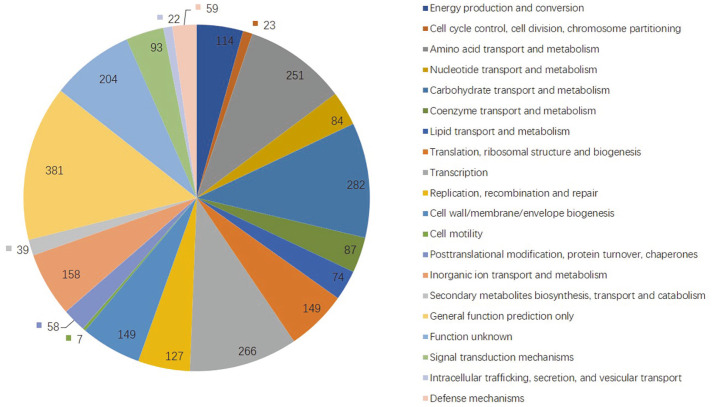
Functional categorization of all predicted open reading frames (ORFs) in the *Lpb. plantarum* HOM3204 genome using the COG database. Diamond (E-value < 1e^-5^) was used for protein alignment.

On the chromosome, 2,247 genes (74.2%) were classified into COG functional categories (Fig. 2). Two hundred and fifty-one genes (11.17%) belonged to amino acid transport and metabolism, 282 genes (12.55%) belonged to carbohydrate transport and metabolism, 266 genes (11.84%) belonged to transcription, and 381 genes (16.96%) belonged to general function prediction only.

### Comparison of *Lpb. plantarum* Strains

**Fig. 3:**
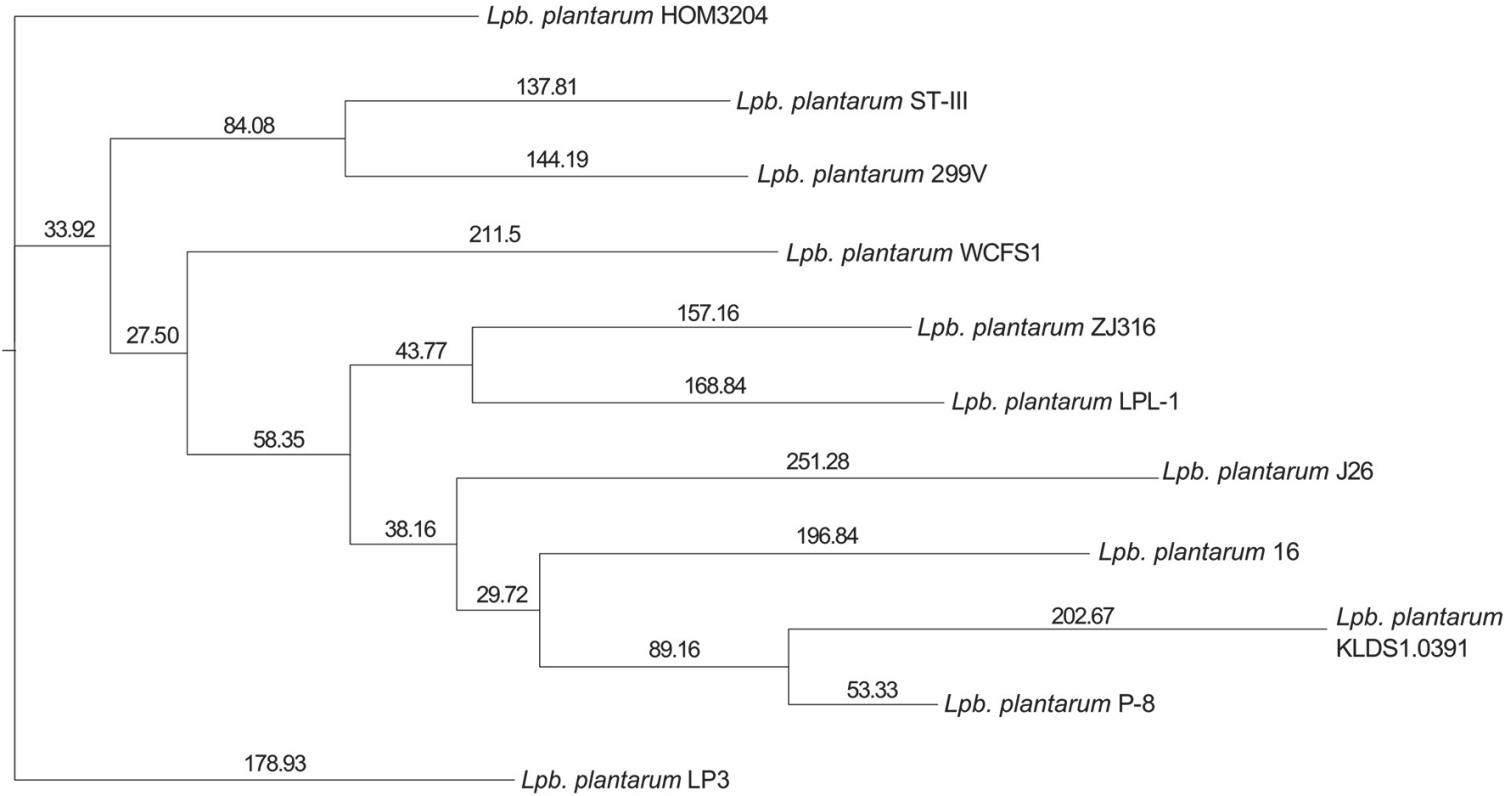
ML tree analysis of *Lpb. plantarum* HOM3204 with 10 available complete genome sequences of *Lpb. plantarum*. ParaAT (V2.0) was used as a parallel tool for constructing multiple protein-coding DNA alignments. The maximum likelihood (ML) phylogenetic tree was constructed using RAxML. Numbers above the branches indicate the bootstrap supports from 500 replicates. The higher the bootstrap value, the more reliable is the evolution tree.

**Fig. 4:**
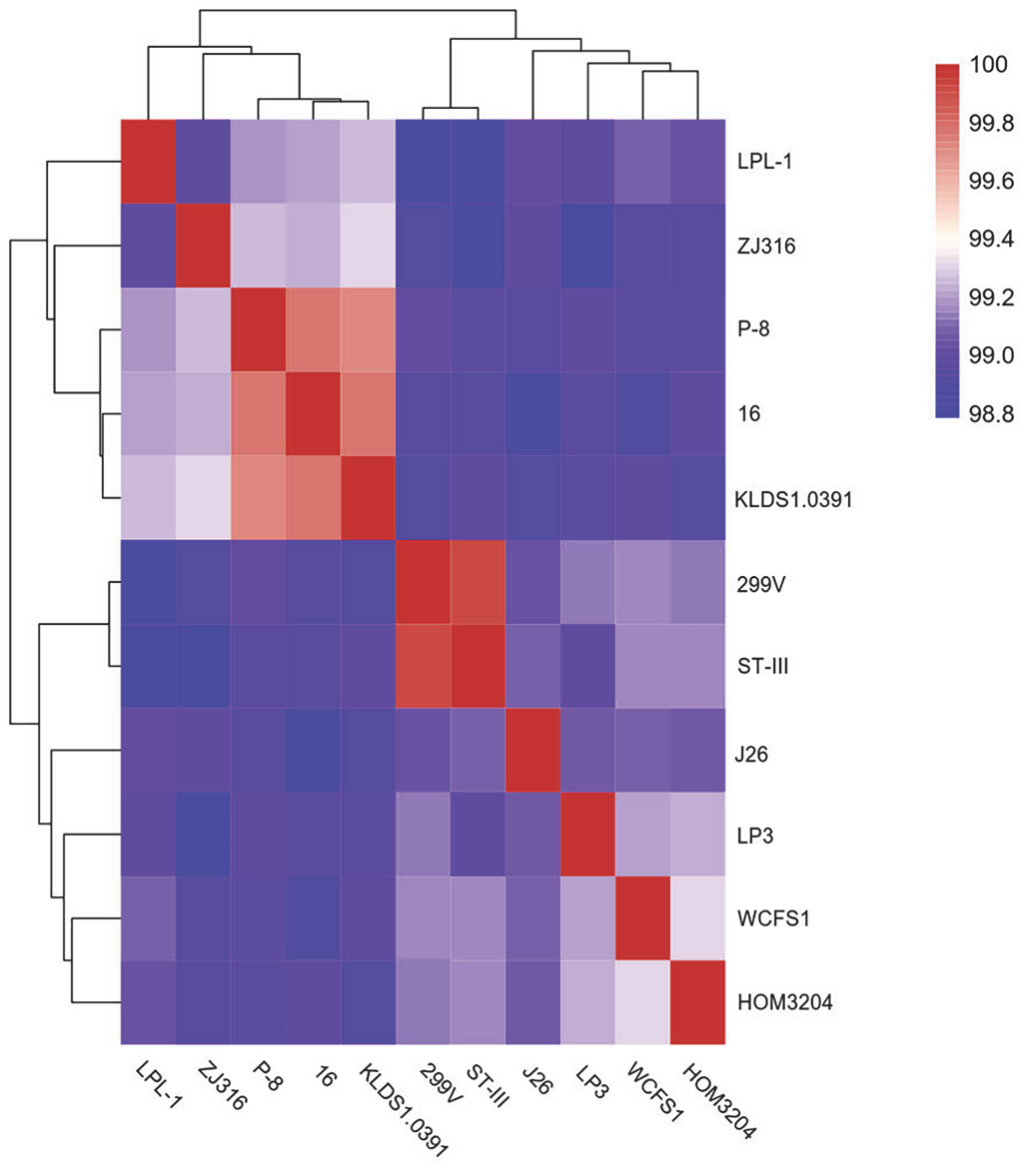
Average nucleotide identity (ANI) tree analysis of *Lpb. plantarum* HOM3204 and 10 available genome sequences of *Lpb. plantarum* strains. ANI tree was constructed using Pyani software.

To understand the evolutionary relationship between the strains, ML and ANI trees were constructed. The results are shown in Figs. 3 and 4, respectively. According to the analysis of the ML tree, 10 strains, namely ST-III, 299v, WCFS1, ZJ316, LPL-1, J26, 16, KDLS1.0391, P-8, and LP3, were not grouped together with HOM3204, suggesting that *Lpb. plantarum* HOM3204 differs from these strains and may have unique features and functions. The ANI tree was built using the same genomes as the ML tree. *Lpb. plantarum* WCFS1 was regarded as the closest neighbor of *Lpb. plantarum* HOM3204 (99.31% of the ANI value).

Genome features of the ten *Lpb. plantarum* reference strains, with detailed WGS information, are presented in Table 2. Each strain had a circular chromosome and a different number of plasmids. The genome size of the control strains was 2.89 to 3.31 Mbp, and the number of plasmids varied from zero to ten.

### Oxidative Stress-Related Proteins

We identified the oxidative stress-related proteins encoded in the genome of *Lpb. plantarum* HOM3204 in the Gene Ontology and COG databases (Table 3). The proteins consisted of GSH-Px, glutathione-disulfide reductase, proteins for removal of superoxide radicals, proteins for removal of oxygen radicals, catalytic proteins, catalase, flavin reductase (NADH), thioredoxin-disulfide reductase, thioredoxin peroxidase, thioredoxin reductase, and a DNA-binding ferritin-like protein named Spy1531. Therefore, the oxidative stress-related proteins in *Lpb. plantarum* HOM3204 can potentially cope with oxidative stress.

### In Vitro Antioxidant Activity

In this study, six indices (T-AOC, hydroxyl radical, DPPH radical, SOD, GSH-Px, and GSH) were chosen to evaluate the antioxidant activity of *Lpb. plantarum* HOM3204 in vitro (Table 4). The intracellular cell-free extracts of *Lpb. plantarum* HOM3204 exhibited the strongest T-AOC activity (23.84 ± 2.44 U/ml) compared to *Lpb. plantarum* ST-III (4.13 ± 0.44 U/ml, *p* < 0.01), *Lpb. plantarum* Lp-115 (15.48 ± 1.13 U/ml, *p* < 0.01), and 0.05%vitamin C (16.90 ± 1.42 U/ml, *p* < 0.01). The DPPH radical scavenging rate of *Lpb. plantarum* HOM3204 (94.18 ± 0.45%) was higher than that of *Lpb. plantarum* Lp-115 (91.03 ± 0.53%, *p* < 0.01) and *Lpb. plantarum* ST-III (93.00± 0.65%, *p* > 0.05), but lower than that of 0.05% vitamin C (96.14 ± 0.08%, *p* < 0.01). *Lpb. plantarum* HOM3204 exhibited the highest SOD activity (28.89 ± 0.30 U/ml) compared to *Lpb. plantarum* ST-III (27.34 ± 0.52 U/ml, *p* < 0.05) and *Lpb. plantarum* Lp-115 (24.63 ± 2.11 U/ml, *p* < 0.05) strains. The GSH content of *Lpb. plantarum* HOM3204 (37.56 ± 2.81 U/ml) was significantly higher than that of *Lpb. plantarum* ST-III (23.72 ± 3.98 U/ml, *p* < 0.01) and lower than that of *Lpb. plantarum* Lp-115 (59.85 ± 5.57 U/ml, *p* < 0.01). The hydroxyl radical-scavenging abilities and GSH-Px activities of the three strains were similar.

### Antioxidant Effect of *Lpb. plantarum* HOM3204 on D-Galactose-Induced Aging in Mice

D-Galactose-induced aging mice showed a significant increase in the level of MDA compared to the control group (D-galactose vs. control, 6.31 ± 0.85 vs. 7.77 ± 1.11 nmol/ml, *p* < 0.01), indicating the successful construction of the oxidative damage model. After 45 days of oral administration of *Lpb. plantarum* HOM3204 powder or sterilized water, there was no significant difference in the body weight between the model and control groups (*p* > 0.05), or between the *Lpb. plantarum* HOM3204 and model groups (*p* > 0.05) (data not shown). The probiotic sample had no adverse effects on the body weight of mice.

The effects of *Lpb. plantarum* HOM3204 in D-galactose-induced aging mice is shown in Table 5. There was no significant difference between the *Lpb. plantarum* HOM3204 group and the model group in MDA, protein carbonyl, and SOD. GSH-Px activity in the whole blood of the *Lpb. plantarum* HOM3204 group was significantly higher than that of the model group (469 ± 68 U/ml vs. 390 ± 83 U/ml, *p* < 0.05). GSH content in the liver tissues of the *Lpb. plantarum* HOM3204 group was significantly higher than that in the liver tissues of the model group (7.35± 1.47 U/ml vs. 6.17 ± 0.79 U/ml, *p* < 0.05). These results demonstrated that the administration of 1 × 10^9^ CFU *Lpb. plantarum* HOM3204 for 45 days alleviated oxidative stress in D-galactose-induced aging mice.

## Discussion

WGS is generally used to study the information and potential functions of genes. Functional genomics research helps to better understand the molecular mechanisms of action of probiotics. [49]. Currently, 682 genome datasets of *Lpb. plantarum* strains are available on the NCBI genome database.

We used the ML and ANI trees for the comparative analysis of *Lpb. plantarum* HOM3204 and 10 reference *Lpb. plantarum* strains. The ML tree showed *Lpb. plantarum* HOM3204 to be a unique strain that differs from the other 10 *Lpb. plantarum* strains. In addition, our ANI tree results revealed *Lpb. plantarum* WCFS1 was the closest neighbor of *Lpb. plantarum* HOM3204 (99.31% of the ANI value), indicating that the two strains share the highest similarity. Originally isolated from human saliva, *Lpb. plantarum* WCFS1 is one of the best-explored model strains and has many good characteristics, such as lowering triglyceride and low-density lipoprotein levels in high-fat diet-induced hypercholesterolemia and hepatic steatosis in mice [11, 50].

Hydroxyl radical, DPPH radical, T-AOC, SOD, GSH-Px, and GSH have been widely used as evaluation indices for ROS-related antioxidant activity [19, 20]. Strains with strong antioxidant activity can cope with oxidative stress. In this study, *Lpb. plantarum* HOM3204 exhibited strong antioxidant activity, as verified by the in vitro and animal experiments. In vitro, the intracellular cell-free extracts of *Lpb. plantarum* HOM3204 exhibited stronger antioxidant properties (*e.g.*, T-AOC and DPPH radical scavenging, SOD) and GSH activities than the reference strain. Oral administration of 1 × 10^9^ CFU *Lpb. plantarum* HOM3204 powder decreased the MDA levels and increased the SOD, GSH-Px, and GSH levels in the serum or liver tissue samples of model mice.

Oxidative stress-related proteins, particularly SOD and GSH-Px, were identified in the genome of *Lpb. plantarum* HOM3204. SOD is an antioxidant enzyme that plays a major role in catalyzing the highly reactive superoxide anion to O_2_ and the less reactive species, hydrogen peroxide (H_2_O_2_) [48]. GSH is an important cellular non-enzymatic antioxidant that is used for reducing lipid peroxides and H_2_O_2_ and catalyzes the conversion of GSH into glutathione disulfide (GSSG) [51]. GSSG is transformed into GSH through the cooperation of GR and NADPH to maintain the GSH redox ratio (GSSG/GSH) [51]. A complete glutathione system comprises the basic components of GSH, GSH-Px, GR, and GSSG [52]. Kullisaar *et al*. showed that L. fermentum ME-3 possesses a complete glutathione system and can transport GSH from the environment to synthesize GSH [51]. The capacity of *Lpb. plantarum* HOM3204 to alleviate oxidative stress may be attributed to its participation in the GSH system. A previous study reported that *Lpb. plantarum* CCFM10 alleviated oxidative stress and restored gut microbiota in D-galactose-induced aging mice. Besides, CCFM10 restored the relative abundance of *Lactiplantibacillus* and suppressed the increase in the abundance of *Clostridiales*. The protective effect on microbiota could be one of the mechanisms of resistance to oxidative stress in vivo [21].

In this study, we proved that *Lpb. plantarum* HOM3204 possesses strong antioxidant activity in terms of T-AOC and SOD, GSH, and DPPH radical scavenging activities in vitro. Moreover, oral administration of *Lpb. plantarum* HOM3204 alleviated oxidative stress in D-galactose-induced aging mice. Our results suggest *Lpb. plantarum* HOM3204 as an effective probiotic with strong antioxidant properties. However, its specific action mechanism needs to be investigated further in future studies.

## Figures and Tables

**Table 1:** Genome features of *Lactiplantibacillus plantarum* HOM3204.

Attribute	Chromosome	Plasmid 1	Plasmid 2
Genome size (bp)	3,232,697	48,573	17,060
DNA GC content (%)	44.61	39.04	40.57
Protein-coding genes	2,247	35	7
rRNA genes	16	0	0
tRNA genes	68	0	0
sRNA genes	38	3	1

**Table 2:** Comparison of the chromosomal properties of different *Lpb. plantarum* strains.

Strain	HOM3204	WCFS1	LP3	ST-III	299v	J26	LPL-1	16	P-8	ZJ316	KLDS1.0391
Genome size (bp)	3,232,697	3,308,273	3,259,858	3,254,376	3,302,055	3,096,468	3,186,859	3,044,678	3,035,719	3,203,964	2,886,607
No. of plasmids	2	3	2	1	0	4	1	10	7	3	3
GC content (%)	44.61	44.47	44.50	44.58	44.40	44.80	44.65	44.74	44.80	44.65	44.80
Annotated genes	3,064	3,116	3,077	3,071	3,153	3,043	3.049	2,874	2,956	3,043	2,891
tRNA genes	72	72	73	70	57	70	67	68	71	63	52
rRNA genes	16	16	16	15	3	16	16	16	16	15	13
ANI (%)	100%	99.31%	99.22%	99.16%	99.14%	99.06%	99.03%	98.98%	98.98%	98.94%	98.93%

**Table 3:** Oxidative stress-related proteins of *Lpb. plantarum* HOM3204.

Oxidative stress-related protein	Locus tag	Database
Glutathione peroxidase	Chr-gene 0184	GO:0004602/COG0386
Removal of superoxide radicals	Chr-gene 0584	GO:0019430
Glutathione-disulfide reductase	Chr-gene 0323, Chr-gene 0991, Chr-gene 1464, Chr-gene 2697	GO:0004362
Response to the oxygen radical	Chr-gene 1464, Chr-gene 2697	GO:0000305
Catalytic activity	Chr-gene 0102, Chr-gene 1140, Chr-gene 1357, Chr-gene 1915, Chr-gene 1931, Chr-gene 2678	GO:0003824
Catalase	Chr-gene 2929	GO:0004096
Flavin reductase (NADH)	Chr-gene 0045, Chr-gene 1116, Chr-gene 2249, Chr-gene 2250, Chr-gene 2680,	GO:0036382
Thioredoxin-disulfide reductase	Chr-gene 0584, Chr-gene 1888, Chr-gene 2175, Chr-gene 2817	GO:0004791
Thioredoxin peroxidase	Chr-gene 1928	GO:0008379
Thioredoxin reductase	Chr-gene 0584, Chr-gene 2138	COG:0492
DNA-binding ferritin-like protein	Plasmid 2-gene 0005	COG:0783

**Table 4:** Antioxidant activities of different *Lpb. plantarum* strains in vitro.

Strain	T-AOC (U/ml)	·OH scavenging (%)	DPPH scavenging (%)	SOD (U/ml)	GSH-Px (U/ml)	GSH (mg/l)
HOM3204	23.84 ± 2.44	76.84 ± 0.36	94.18 ± 0.45	28.89 ± 0.30	20.57 ± 2.73	37.56 ± 2.81
ST-Ⅲ	4.13 ± 0.44**	76.57 ± 0.36	93.00 ± 0.65	27.34 ± 0.52*	24.29 ± 3.18	23.72 ± 3.98**
Lp-115	15.48 ± 1.13**	77.16 ± 0.60	91.03 ± 0.53**	24.63 ± 2.11*	15.60 ± 2.06	59.85 ± 5.57**
Vitamin C	16.90 ± 1.42**	36.70 ± 1.33**	96.14 ± 0.08**	34.32 ± 0.36**	ND	ND

Comparison of the *Lpb. plantarum* HOM3204 strain with other strains: *p < 0.05, **p < 0.01. ·OH, hydroxyl radical scavenging.

ND, not determined.

**Table 5:** Effects of *Lpb. plantarum* HOM3204 on malonaldehyde (MDA), protein carbonyl, superoxide dismutase (SOD), glutathione peroxidase (GSH-Px), and glutathione (GSH) levels in the D-galactoseinduced oxidative injury mouse model.

Group	MDA (nmol/ml)	Protein carbonyl (nmol/mgprot)	SOD (U/ml)	GSH-Px (U/ml)	GSH (mgGSH/gprot)
Model group	7.81 ± 1.43	7.02 ± 1.36	213 ± 38	390 ± 83	6.17 ± 0.79
HOM3204	7.59 ± 1.19	6.49 ± 1.98	230 ± 48	469 ± 68*	7.35 ± 1.47*

Comparison of the *Lpb. plantarum* HOM3204 group with the model group: *p < 0.05.
